# Metabolomic Approach to Study the ‘Purple Queen’ Pomegranate Cultivar Response to Alternative Culture Media and Phenological Stages

**DOI:** 10.3390/foods12020352

**Published:** 2023-01-11

**Authors:** Juan José Martínez-Nicolás, Francisca Hernández, Dámaris Núñez-Gómez, Francisco García-Sánchez, Rafael Martínez-Font, Pilar Legua, Pablo Melgarejo

**Affiliations:** 1 Centro de Investigación e Innovacion Agroalimentaria y Agroambiental (CIAGRO-UMH), Miguel Hernandez University, Ctra. Beniel km 3.2, 03312 Orihuela, Spain; 2 Centro de Edafologia y Biologia Aplicada del Segura, Consejo Superior de Investigaciones Científicas, Campus Universitario de Espinardo, Espinardo, 30100 Murcia, Spain

**Keywords:** *Punica granatum*, port sediment, metabolomic differentiation, multivariate analysis, 1H-NMR

## Abstract

The increasingly evident threat of depletion of world peat bogs is encouraging the search for and study of alternative agricultural substrates that can fully or partially replace peat, guaranteeing food supply (quality and quantity). On the other hand, the identification of the potential for the reuse of waste from relevant economic activities has increased in recent years, mainly motivated by the change to a sustainable circular economy, as is the case of port sediments. Taking into account that significant volumes of dredged port sediments are generated annually so that ports can maintain their economic activity, it is necessary to find objective, sustainable and safe reuse alternatives. In this sense, the objective of this study was to study the response of the “Purple Queen” pomegranate when grown with dredged port sediment. For this, the fruit production (kg), number of fruits (fruits tree-1), fruit weight (g), and seed yield (%) aiming to verify the correct tree development were evaluated. In addition, a 1H-NMR foliar metabolomic study for the three most relevant phenological phases was performed (flowering, fruit development, and post-harvest) to identify metabolic changes in trees. In total, 29 metabolites were identified; among them, 11 were amino acids, 6 organic acids, 5 sugars, and 7 secondary metabolites. The good agronomical development of the trees and fruits indicated the potential for using the dredged sediment as an agricultural substrate. On the other hand, the results revealed that the greatest variability in the metabolomic study occurred between the phenological phases and a lower variability is explained by the substrates used.

## 1. Introduction

Large amounts of sediment are dredged from seaports every year, aiming to maintaining their depths and, therefore, their activity [1]. These operations directly or indirectly generate negative impacts on the environment, which can generate physical, chemical, and biological changes in ecosystems characteristics [1,2]. These impacts may be due to the dredging process itself, the sediment excavation/extraction, material loss during its transport to the surface and/or coast, or their transfer to safe disposal places with environmental limitations [3]. Currently, European legislation classifies the dredged sediments as residue, restricting their use since they may present contaminants derived from port transit and its activities [4]. Nevertheless, aware of the need to carry out the periodic dredging of port sediments and their impacts, the EU has been looking for alternative, economically and ecologically sustainable solutions that allow their integrated management with the objectives of the circular economy, highlighting the worth of more than 30 LIFE projects focused on the study of both the remediation of sediments and the identification of safe alternatives for their reuse, principally as agricultural substrates [3,5,6,7].

During the last five decades, the most used substrate for plant cultivation has been peat [8], since it represents an ideal culture medium due to its physicochemical characteristics, optimal for many plant species and for the management of different farming systems [9,10]. However, the pillaging of these peatlands around the world over the years can pose a problem of depletion, since it is a limited resource [11]. For this reason, its necessary to obtain new substrates allowing us to reduce or even replace the use of peat in agriculture. Numerous studies around the world indicate the potential revalorization and adequacy of the use of some waste and/or by-products as a substrate to replace peat [12,13,14,15,16]. Studies on dredged sediments have shown that decontamination techniques, such as phytoremediation, can be successful in obtaining new substrates for plant growth [7,17]. This phytoremediation is considered the most cost-effective and environmentally friendly soil reclamation strategy, constantly developing to become an effective and reliable remediation method for sediments contaminated by a wide range of pollutants [18]. Despite all this, the use of these sediments in agricultural uses still affects small volumes of material; in this context, the general objective of this study was to study the suitability of the remediated dredged sediments, alone and mixed with peat, as a traditional substrate alternative. To verify its suitability as a culture substrate for the growth of woody plants, pomegranate trees (*Punica granatum* L. cv “Purple Queen”) were used. The pomegranate was chosen because it is one of the most important emerging fruit trees in the world, with a significant increase in its demand in recent decades both for fresh consumption and industrially processed products (juices, alcoholic beverages, jams, dehydrated seeds, nutritional fiber, dried bark to make infusions and extracts from its different parts) but also in other industries as cosmetic, pharmaceutical, and medical. This growing interest in pomegranate fruits can be motivated by their high bio compounds content and their beneficial impacts on human health [19].

In addition to the traditional agronomic study (production, morphological characteristics of the fruit, etc.), when alternative cultivation substrates are used, the identification and investigation of their impact on plant metabolism are recommended. Since the content of compounds in plants is very complex, performing a metabolomic analysis to predict the plant’s response is becoming more and more necessary. Metabolomics can also be used to predict marker compounds that play a role in a particular activity [20] and has been widely used in plant science as physiology, stress tolerance, phytochemistry, flavor studies, and food technology [21,22,23]. Therefore, the knowledge and identification of the metabolomic profile can indicate the response of the plant to changes in the cultivation conditions, or as in this study, the use of an alternative substrate to peat (dredged sediment).The objective of this study was to characterize the behavior of the “Purple Queen” pomegranate cultivar grown in peat and in a phytorremediated port substrate and identify its impacts during three phenological stages (flowering and fruit set, fruit development, and post-harvest), according to their metabolomic composition. To achieve this objective, an agronomic study (vegetative growth, production, and quality of the fruit) was carried out followed by leaf metabolomic analysis for each phenological phase, aiming to ascertain which compounds are the most demanded in each phenological stage and if these compounds are related to the agronomic characteristics of the culture media used.

## 2. Materials and Methods

### 2.1. Plant Material and Experimental Design

The trial was developed in an experimental crop plot at the Higher Polytechnic School of Orihuela of Miguel Hernandez University (Southeast of Spain, 38°04′ N, 0°58′ W, 26 m above sea level). Pomegranate trees (*Punica granatum* L., cv “Purple Queen”) were cultivated in 40 L polyethylene pots using three different substrates: (1) 100% peat (PS0) as a substrate control; (2) 100% dredged remediated sediments (PS100); and (3) 50% peat and dredged remediated sediment mixed (PS50). The dredged remediated sediments (PS100) used came from Livorno Port (Italy) and was previously phytoremediated for three years [24,25]. The PS100 characteristics and composition were already reported by the authors [26,27].

For each substrate, 15 pomegranate trees (3 blocs distributed using a completely randomized block design × 5 trees per block) were used. In addition to the total 45 pomegranate trees employed in the experiment (3 substrates × 3 blocs × 5 trees per bloc), 40 trees were further cultivated with peat and placed surrounding the treated trees in order to avoid the border effect. The crop management was homogeneous for all the trees and according to described by Melgarejo et al. [26]. All the trees were present a good phytosanitary condition and correct development both during the time of the study and sample collect.

### 2.2. Plant Samples and Physicochemical Determinations

Foliar sampling was carried out in three critical phenological stages for the pomegranate crop: full flowering and fruit set (FP); fruit development (DF); and postharvest (PostR). In each phenological moment, 18 leaf samples were manually taken (3 substrates × 3 blocs × 2 leaves). The leaves samples were processed and storage according to [28]. The fruit production (kg), number of fruits (fruits tree^−1^), fruit weight (g), and seed yield (%) were also studied and monitored as a way to controlled and verified the correct tree development.

At the end of the experimental crop, the two-year-old pomegranate trees were sacrificed and divided into plant parts: root, stem, and leaves. The pomegranate plant parts samples were weighed separately and oven-dried (Model ED23, Binder Inc., Tuttlingen, Germany) at 60 °C till constant weight. The total biomass was determined using the data obtained from biomass of the aerial part (leaves and stem) and root biomass both in dry form (g dw^−1^).

Before the tree sacrifices, the tree height and leaf surface were also determined, with a tape measurement and leaf area meter (Area Meter Licor Li3100), respectively. In addition, the leaf color was studied using a colorimeter (CM-700d spectrophotometer colorimeter) through the L*, a*, and b* parameters [29]. For the determination of the total chlorophyll content of the biomass, the spectrophotometric method proposed by [30] was used.

### 2.3. Metabolomic Analysis

A directed metabolic study of leaf samples was carried out. After collection, the leaf samples were stored at −80 °C, no more than 12 h, until their lyophilization, aiming to maintain their metabolites composition. Afterwards, the extraction was carried out following the protocol of [31] with some modifications as described in [28]. The analysis was carried out with an Ascend 500 MHz AVANCE III HD H-NMR (Bruker Scientific Instrument, Heidelberg, Germany). The resulting spectra were evaluated with the “Chenomx NMR Suite” Program version 8.3. A detailed description of the method can be found in [28].

### 2.4. Statistical Analysis and Metabolic Pathway Analyses

Statistical analyses were performed using SPSS 25.0 for Windows (SPSS Science, Chicago, IL, USA). A basic descriptive statistical analysis was followed by an analysis of variance (ANOVA) test for mean comparisons. The method used to discriminate among the means (multiple range test) was Tukey’s HSD test (*p* ≤ 0.05). A principal component analysis (PCA) has been carried out for the three substrates tested, based on the results of the metabolomic analysis of the leaves, in the same way another PCA was carried out with these same analyses for the three phenological stages considered.

In addition, the identification and definition of partial least squares discriminant analysis (PLS-DA) and variable importance in projection (VIP) scores were also determined by specific software for metabolomic data processing, MetaboAnalyst 5.0 (Wishart Research Group, University of Alberta, Edmonto, Canada), which allowed us to determine metabolites contributing to significant between-group differences [32].

## 3. Results

### 3.1. Agronomical and Vegetative Characterization

The physical parameter results for the pomegranate trees and fruits are shown in Table 1. A significant difference in the average production per tree, being between 30 and 35% lower in trees grown on PS100, in relation to those grown on PS0 and on PS50. The same tendency was observed for the average weight of the fruits, which is 8% higher in the mixture than in PS100, and 13% higher in PS0 than in PS100.

The content of chlorophylls a, b, and a + b is significantly higher in trees grown in peat than in sediment, not appreciating significant differences with respect to the mixture, which presents intermediate levels between PS0 and PS100. The weight of the aerial part was 70% higher in PS0 than in PS100, and 57% higher in PS50. The same trend was maintained in the root, but with differences between peat, sediment, and mixture of 41 and 22%, respectively. Regarding the total weight of the tree, it was 67% higher in peat than in the sediment and 62% higher than in the mixture. Regarding the pomegranate fruits, the only significant difference found is the one relative to the size, which was higher in the culture in peat compared to the culture in PS100 (Table 1).

### 3.2. Leaves Metabolomic Analysis

For all the samples, 29 compounds were identified, among which 11 were amino acids, 11 were organic acids, and 7 were secondary metabolites and others. Table 2 shows the compounds obtained in the metabolomic analysis, organized into three homogeneous groups (amino acids, organic acids and sugars and secondary metabolites and others). The two independent variables (substrate and phenological stage) have been considered, as well as their interactions. Statistical significance differences for each compound related to the independent factors are indicated with a * in Table 2. It can be highlighted that the phenological stage was significant for almost all the compounds obtained from the analysis, except for alanine and aspartate. For the substrate variable, only 4 amino acids (GABA, arginine, leucine, and tryptophan), 4 organic acids (succinate, citrate, fructose, and glucose) and 2 secondary metabolites (choline and ferulate) were identified as significant. The substrate × phenological stage interaction was significant for tryptophan, glutamine, acetate, malate, citrate, sucrose, fructose, and glucose. This shows that the content of each of these metabolites depends on the combination of substrate × phenological stage that is analyzed.

The ANOVA, and the following contrast of means carried out with the Tukey test for *p* < 0.05, for the results of the metabolomic analysis based on the culture substrate factor, indicates that this factor significantly influenced the amino acids GABA, arginine and tryptophan, in glucose and in ferulate, epicatchin, and vanillate (Table 3). In the mixture and in the sediment, the three indicated aa presented higher values than those obtained in peat. Glucose was higher in the mixture than in peat, but without significant differences regarding the sediment. Regarding secondary metabolites, ferulate was significantly higher in sediment and mixture than in peat, while for epicatechin and vanillate it was the opposite.

Related to the phenological stages evaluated, glutamate showed the highest values in the flowering and fruit development periods, formate in the fruit development, acetate and malate in the flowering period, fumarate in the fruit development and postharvest periods, while citrate, sucrose, fructose, and glucose had higher values in the flowering and postharvest periods. In the group of secondary metabolites, it is observed that the highest values of choline, trigonelline, and adenine occur during the flowering period, while in gallate the highest levels occurred in the periods of fruit development and post-harvest (Table 4).

The component analysis of variance (Table 5) indicates that the weight of the phenological stage factor over the concentration of the compounds determined by metabolomic analysis predominates over the effect of the substrate factor in most amino acids, in all organic sugars and acids, and in most of the secondary metabolites and others. The influence of the substrate was greater in alanine, valine, ferulate (86.45%), epicatechin (70.72%), and vanillate (71.38%).

### 3.3. Multivariate Statistical Analysis

#### 3.3.1. Principal component analysis (PCA) by substrates

##### Amino Acids (AA)

**Figure 1:**
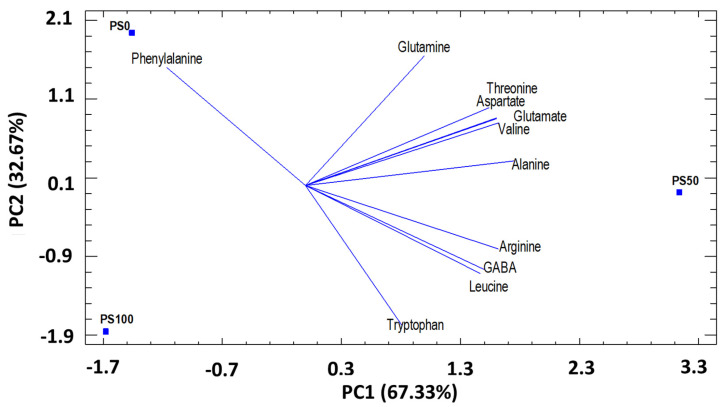
Principal components analysis (PC1 and PC2) for amino acids identified in pomegranate leaves cultivated in three different substrates. PC1 (67.33%) and PC2 (32.67%).

To achieve a better understanding of the trends and relationships between the amino acids studied (11) in pomegranate leaves (in three substrates), principal component analysis (PCA) was applied. The first two principal components (PC) explained 100% of the total variation. The first component (PC1), which represents 67.33% of the total variance, was related to the amino acids’ GABA, alanine, valine, arginine, leucine, threonine, aspartate, and glutamate. PC2 represented 32.67% of the total variance. It was correlated with the content of tryptophan, phenylalanine, glutamate (Figure 1). The PCA results showed that the PC1 axis allows to clearly discriminate the PS0 and PS100 substrates from the PS50 substrate (Figure 1). PC2 allows discrimination of PS0, PS50 and PS100 substrates, especially PSO of PS100.

##### Sugars and Organic Acids

**Figure 2:**
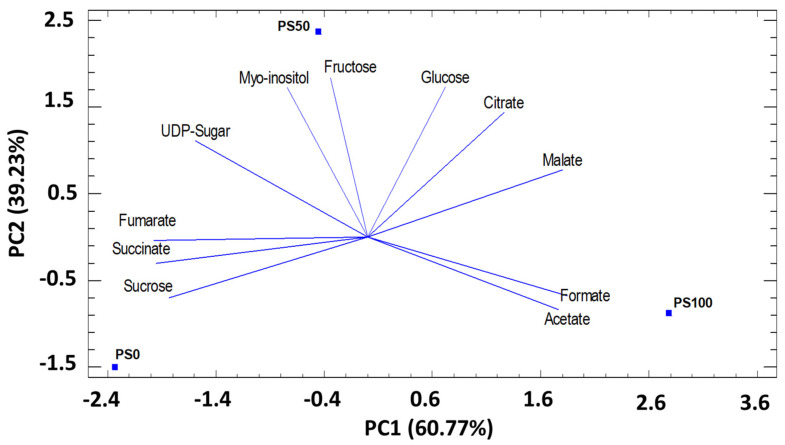
Principal components analysis (PC1 and PC2) for sugar and organic acids identified in pomegranate leaves cultivated in three different substrates. PC1 (60.77%) and PC2 (39.23%).

Regarding PCA for sugars and organic acids studied (11) in pomegranate leaves (in three substrates), the first two principal components (PC) explained 100% of the total variation. The first component (PC1), which represents 60.77% of the total variance, was related to the organic acids formate, acetate, malate, fumarate, succinate and with the sugar’s sucrose, fructose, glucose, and UDP-sugar. PC2 represented 39.23% of the total variance. It was correlated with the content of citrate, fructose, glucose, myo-inositol (Figure 2). The PCA results showed that the PC1 axis allows to discriminate the PS0 and PS50 substrates from the PS100 substrate, and especially PS0 from PS100. PC2 allows discrimination of PS50 substrates from PS0 and PS100 (Figure 2).

##### Secondary Metabolites and Others

**Figure 3:**
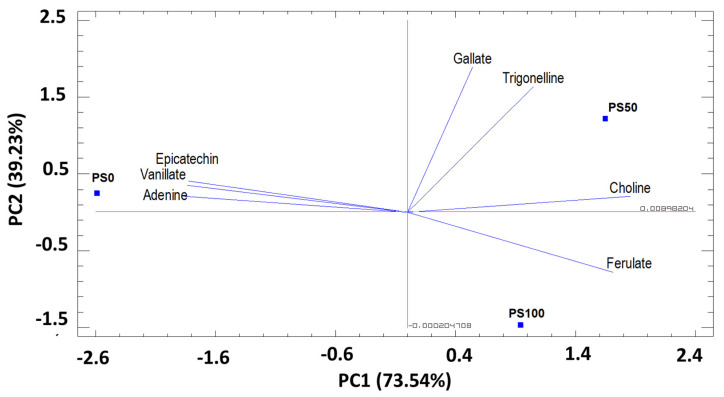
Principal components analysis (PC1 and PC2) for secondary metabolites identified in pomegranate leaves cultivated in three different substrates. PC1 (73.54%) and PC2 (26.46%).

The PCA of secondary metabolites and others (7 metabolites) in pomegranate leaves (on three substrates), confirmed that the first two principal components (PC) explained 100% of the total variation. The first component (PC1), which represents 73.54% of the total variance, was related to choline, adenine, ferulate, epicatechin, and vanillate. PC2 represented 26.46% of the total variance. It was correlated with the content of trigonelline and gallate (Figure 3). The PCA results showed that the PC1 axis allows to discriminate the PS0 substrate from the PS50 and PS100 substrates (Figure 3). PC2 allows discrimination of PS0, PS50, and PS100 substrates.

#### 3.3.2. Principal Component Analysis (PCA) by Phenological Stage

##### Amino Acids (AA)

**Figure 4:**
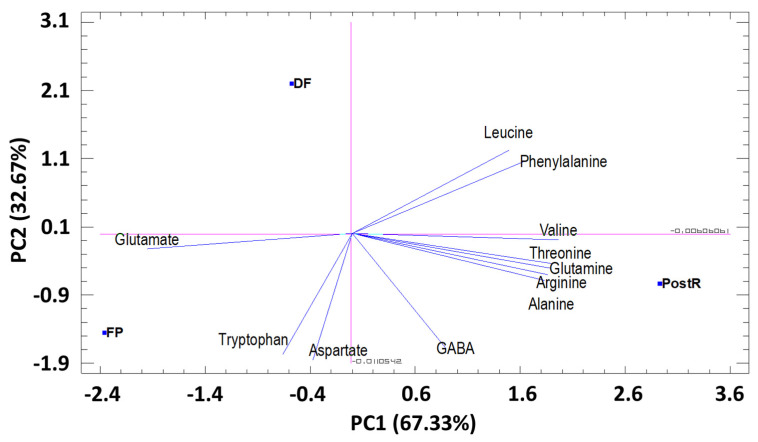
Principal components analysis (PC1 and PC2) for amino acids identified in pomegranate leaves cultivated in three different phenological stages. PC1 (65.85) and PC2 (34.16%).

To achieve a better understanding of the trends and relationships between the amino acids studied (11) in pomegranate leaves (in three phenological stages), principal component analysis (PCA) was applied. The first two principal components (PC) explained 100% of the total variation. The first component (PC1), which represents 65.85% of the total variance, was related to the amino acids, alanine, valine, arginine, threonine, phenylalanine, glutamine, and glutamate. PC2 represented 34.16% of the total variance. It was correlated with the content of GABA, leucine, tryptophan, and aspartate (Figure 4). The PCA results showed that the PC1 axis allows to discriminate the phenological stages FP and DF of PostR. PC2 allows discriminating the phenological stage DF from the phenological stages FP and PostR (Figure 4).

##### Sugar and Organic Acids

**Figure 5:**
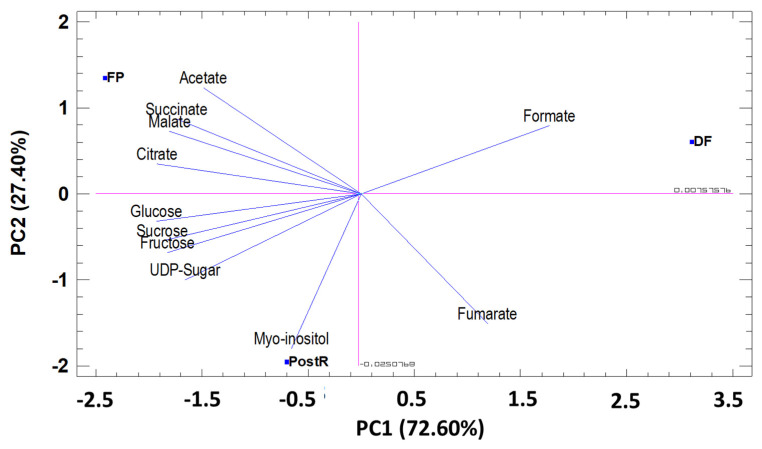
Principal components analysis (PC1 and PC2) for sugar and organic acids identified in pomegranate leaves cultivated in three different phenological stages. PC1 (72.60%) and PC2 (27.40%).

The PCA for the sugars and organic acids studied (11) in pomegranate leaves (in three phenological stages), showed that the first two principal components (PC) explained 100% of the total variation. The first component (PC1), which represents 72.60% of the total variance, was related to formate, malate, succinate, citrate, sucrose, fructose, glucose, and UDP-sugar. PC2 represented 27.40% of the total variance and was correlated with the content of acetate, fumarate and myo-inositol (Figure 5). The PCA results showed that the PC1 axis allows to discriminate the phenological stages FP, PostR and DF. PC2 Allows discriminating the phenological stage DF from the phenological stages FP and PostR (Figure 5).

##### Secondary Metabolites and Others

**Figure 6:**
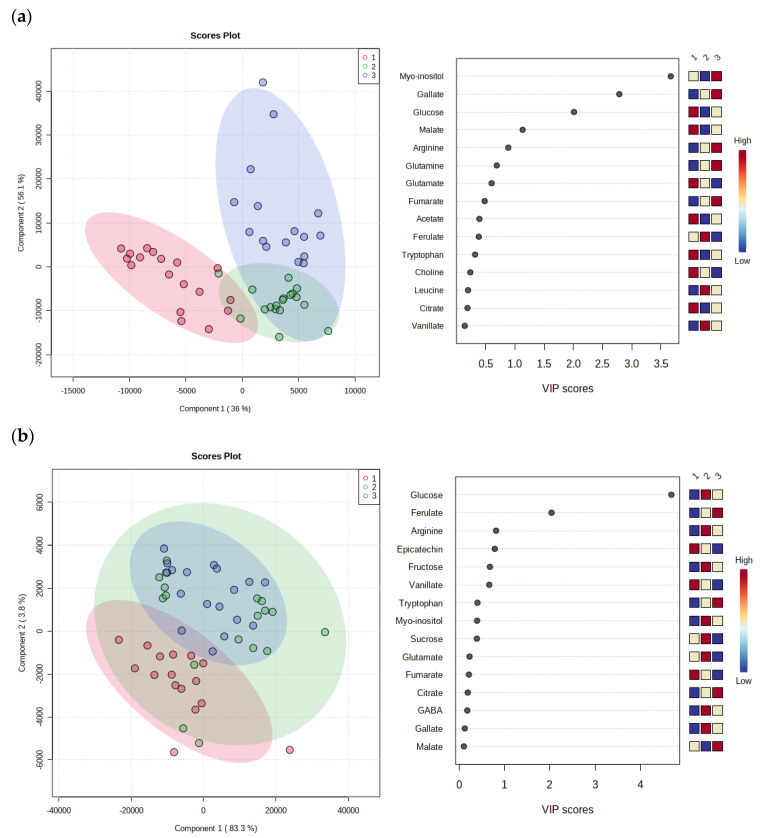
PLS-DA plot for pomegranate tree leaves cultivated in tree substrates and VIP scores with the corresponding heat map where red and blue indicates the level of metabolites. The results were analysed according to (**a**) the phenological stage where 1 represent flowering stage (FP); 2 fruit development (DF); and 3 post-harvest (PostR); and (**b**) the substrate used to pomegranate cultivation where 1 is 100% peat (PS0); 2 peat and sediment mixed (PS50); and 3 100% sediment.

Related to the secondary metabolites, the first two principal components (PC) explained 100% of the total variation. The first component (PC1), which represents 80.46% of the total variance, was related to choline, trigonelline, adenine, gallate, and vanillate. PC2 represented 19.54% of the total variance, it was correlated with the content of ferulate and Epicatechin and (Figure 6). The PCA results showed that the PC1 axis allows to discriminate the phenological stages FP from DF and PostR. PC2 allows us to discriminate DF and PostR (Figure 6).

#### 3.3.3. PLS-DA Regression

A PLS-DA regression was carried out to identify the correlations between the variables studied (substrates and phenological stages). PLS is a supervised method that uses multivariate regression techniques to extract, via linear combination of original variables (X), the information that can predict the class membership (Y). To assess the significance of class discrimination, a permutation test was performed. In each permutation, a PLS-DA model was built between the data (X) and the permuted class labels (Y) using the optimal number of components determined by cross validation for the model based on the original class assignment. In the PLS-DA model generated for the phenological stages the differences between the FP and PostR phenological stages are clearly observed, but both are related to the phenological phase of fruit development (Figure 6a). For the substrates, a differentiation between the PS0 and PS100 was observed, but the PS50 is shown to be broader with all substrates (Figure 6b). In addition, the study of the variable importance projection (VIP) calculated from the PLS-DA analysis confirmed the identification of the myo-inisitol, gallate, glucose and malate as a significant and differentiation between phenological stages, and the glucose, ferulate and arginine metabolites between the substrates used.

## 4. Discusion

The growth parameters of the pomegranate trees and their fruits were between 30 and 35% lower when they are grown on PS100, this is due to the heavy texture of the sediment, which facilitates waterlogging and poor aeration to the root system. For this reason, when mixed with 50% peat (aeration improver) there is an increase in production and fruit size to levels similar to peat cultivation (reference substrate). The same occurs for the chlorophyll content. This sediment behavior was already described for strawberry (*Fragaria* × *ananassa* Duch. Camarosa and Monterey cultivars) and lemon (*Citrus limon* Brum Verna cultivar) [6,27].

### 4.1. Metabolite Profile According to Substrates

The substrate used was significant for the concentration of amino acids GABA, arginine, leucine and tryptophan. For these four amino acids, the lowest concentration occurred in the leaves of trees grown in peat. Villa-Ruano et al. [33] in a study of the nutritional quality of pomegranate juice, in which using a metabolomic approach based on 1H NMR, compared between conventionally and organically grown fruits, and the results revealed that the pomegranate juice obtained from organically grown plants contained higher amounts of acetic acid, alanine, arginine, fumaric acid, GABA, galactose, glutamine, histidine, isoleucine, lactic acid, leucine, malic acid, mannose, methionine, phenylalanine, proline, sucrose, threonine, trigonelline, tyrosine, and valine than obtained from conventionally grown fruits. This is in line with the results shown in this work, save the important differences between the two studies, since in our case we have not worked with fruits but with leaves.

Regarding organic acids and sugars, succinate, citrate, fructose and glucose were significant, showing significantly lower values in general in peat than in the other substrates. These types of results have also been reported by Moradi et al. [21] in a study with drought stress tolerant plants, and the results identified the significantly affected metabolites. These metabolites belonged to different chemical classes (amino acids, carbohydrates, organic acids, and lipids). These compounds may play an important role through different mechanisms including osmotic adjustment, scavenging of ROS (reactive oxygen species), protection of cellular components, and changes in membrane lipids, etc. For secondary metabolites and others, the substrate factor was significant for choline, ferulate, epicatechin, and vanillate, presenting the first two the lowest values in peat and the last two the highest values in the sediment or in the mixture of it with peat at the same proportion (50%).

The glucose and ferulate metabolites identified as significant in the VIP analysis (VIP > 1) would confirm the impact of the culture medium on plant growth since both compounds are related to the development of the cell wall and the use of polysaccharides [34,35].

### 4.2. Metabolite Profile According to Phenological Phases

All the amino acids, except GABA and aspartate, were significant for the phenological stage of the pomegranate trees. Alanine, valine, arginine, leucine, phenylalanine and glutamine were predominant especially in the PostR phenological stage. The highest values of tryptophan and aspartate occurred in the flowering period. Leucine showed high values during the fruit development period.

Within the family of compounds associated with pentose phosphates and tricarboxylic acid pathways, H-NMR detected and quantified fructose, glucose, sucrose, malate, citric acid, fumarate, glutamate, and formate in the different phenological phases. In all the phenological phases studied, we found that the majority sugar was glucose, followed by fructose and sucrose, reaching its maximum concentrations in the flowering and postharvest phases. However, Zhang et al. [36] observed a different pattern in the accumulation of sugars in the leaves of tomato varieties “606” and “112”, where the concentration followed a decreasing order: sucrose > glucose > fructose. These sugars are important in plants because they act as substrates in the metabolic pathways associated with CO_2_ fixation and the transport of carbon and energy to sink organs [37]. In our study, it was observed that there was a significant decrease in the concentration of sugars when passing from the phenological phase of flowering to fruit development, since the fruits demanded a large amount of sugars during their maturation [38]. H-NMR analysis also detected the organic acids malate, citric acid, fumarate, glutamate, and formate. The first three acids participate in the Krebs cycle, or tricarboxylic acid cycle, and the last one (formate) in the glycolic acid pathway [39]. For the organic acids of the Krebs cycle, the most abundant was malate followed by citrate, reaching its highest concentrations in the flowering phase. Malate, in many plants, is the most accumulated acid and performs many functions in plant cells, one of which is its role as an osmolyte and anion, which compensates for the positive charge of potassium, being particularly important in stomata regulation [40]. These data coincide with the results observed in tomato plants varieties “606” and “112”, in which the highest values of malate occurred in the flowering period [36].

Glutamate showed the highest values in the flowering and fruit development periods, formate in the fruit development, acetate and malate in the flowering period, fumarate in the fruit development and postharvest periods, while citrate, sucrose, fructose, and glucose were more abundant in the flowering and postharvest periods.

In the group of secondary metabolites, it is observed that the highest values of choline, trigonelline, and adenine occur during the flowering period, while in gallate the highest levels occurred in the periods of fruit development and post-harvest.

For GABA, aspartate, ferulate, epicatechin, and vanillate, no significant differences were observed between the three phenological phases sampled.

In the phenological stage of flowering, tryptophan, aspartate, glutamate, acetate, malate, citrate and glucose were especially abundant. In the phenological stage of fruit development, phenylalanine, leucine, glutamate, formate, fumarate, and gallate predominated, while in the post-harvest phase, alanine, valine, arginine, leucine threonine, phenylalanine, glutamine, fructose, glucose, UDP-sugar, myoinositol, ferulate, epicatechin, and vanilla predominated.

The metabolites glutamate, glutamine, and aspartate play a key role in the physiological and metabolic processes of plants, since they are involved in the assimilation of ammonium, synchronizing it with metabolic pathways related to plant nutrition, energy, photosynthesis, and responses to abiotic and biotic stresses, in addition to their functions as nitrogen providers, and also participate in the synthesis of the rest of the amino acids [41,42]. Therefore, glutamate in plants could increase their production, and facilitate their adaptation to adverse environmental conditions, such as drought, salinity, etc. [43]. The exogenous application of glutamine to onion plants increased their height, the number and dry weight of the leaves, the length, diameter, and weight of the bulb, as well as their agronomic performance and the quality of the bulbs [44]. Phenylalanine is an aromatic amino acid that is synthesized via the arogenate shikimate pathway. It is also associated with the metabolism of phenylpropanoids, which play an important role in defense against pathogens, protection against abiotic stress, signal transduction, and communication with other organisms [45,46].

## 5. Conclusions

In this work, the behavior of three substrates (peat, marine sediment, and a 50% mixture of both) is studied in pomegranate (*Punica granatum* L., cvr. “Purple Queen”) in three phenological stages (full flowering, fruit development, and postharvest). A metabolomic analysis of the leaves of the trees cultivated in the three substrates and in the three indicated phenological stages was carried out. These studies revealed that the greatest variability in the metabolomic study occurred between the phenological phases and a lower variability is explained by the substrates used. The substrate did not affect the growth of the plants, but the marine sediment used without mixing decreased the production and the size of the fruits. The amino acids that were affected by the type of substrate were GABA, arginine and tryptophan, among the organic acids and sugars citrate, fructose, and glucose were influenced. In the block of secondary metabolites and others, the substrate conditioned the concentration of choline, ferulate, epicatechin, and vanillate.

From the results it can be deduced that the highest values of alanine, valine, arginine, leucine, phenylalanine and glutamine occur in the postharvest phenological stage. The highest values of tryptophan and aspartate in the flowering period. Leucine also showed high values during the fruit development period. The analyses carried out allow us to affirm that the bioremediated marine sediment mixed with peat is suitable for the cultivation of plants.

## Figures and Tables

**Table 1:** Characteristics measured in the pomegranate trees and production obtained for the three substrates tested where PS0 represents the 100% peat; PS50 the mixture of peat and port sediment and PS100 the dredged remediated sediments. The values are the mean ± SE.

	Culture Media		
Parameter	PS0	PS50	PS100
Leaf Surface (cm^2^)	6.77 ± 0.55 a	6.45 ± 0.82 a	5.24 ± 0.49 a
L*	35.75 ± 0.41 a	36.86 ± 0.43 a	37.90 ± 0.38 a
a*	−7.14 ± 0.22 a	−7.24 ± 0.23 a	−7.40 ± 0.17 a
b*	13.46 ± 0.62 a	14.01 ± 0.44 a	14.60 ± 0.46 a
Chroma	15.27 ± 0.63 a	15.79 ± 0.47 a	16.38 ± 0.48 a
Tree height (mm)	211.09 ± 9.50 a	193.92 ± 8.74 a	186.85 ± 8.40 a
Pruning weight	297.95 ± 25.19 a	404.0 ± 30.60 a	343.4 ± 27.49 a
Yield tree^−1^ (kg)	6.63 ± 0.35 a	6.29 ± 0.28 a	4.30 ± 0.37 b
Fruit tree^−1^	22 ± 1.33 a	25 ± 1.29 a	18 ± 1.86 a
Fruit weight (g)	261.8 ± 11.32 a	248.8 ± 11.64 a	227.4 ± 10.29 b
Seed yield (%)	54.22 ± 0.80 a	56.21 ± 1.44 a	52.17 ± 1.43 a
Dry matter (g)	36.36 ± 2.18 a	40.4 ± 2.26 a	38.38 ± 2.18 a
Chlorophyll a	39.39 ± 2.09 a	35.35 ± 1.87 a	25.25 ± 1.33 b
Chlorophyll b	20.2 ± 1.07 a	22.22 ± 1.09 ab	17.17 ± 0.99 b
Chlorophyll a + b	59.59 ± 3.16 a	57.57 ± 3.04 a	42.42 ± 2.25 b
Aerial part weight (g)	7.58 ± 0.42 a	3.23 ± 0.18 b	2.32 ± 0.12 c
Root weight (g)	0.51 ± 0.03 a	0.40 ± 0.02 b	0.30 ± 0.02 c
Total tree weight (g)	8.08 ± 0.46 a	3.64 ± 0.25 b	2.63 ± 0.15 c
*Pomegranate fruits*			
Diam1 equal (mm)	81.88 ± 1.18 a	79.93 ± 1.48 a	78.30 ± 1.34 a
Diam2 chalice (mm)	25.16 ± 0.89 a	20.43 ± 0.89 b	16.48 ± 0.56 b
Long1 fruit (mm)	73.91 ± 1.16 a	70.05 ± 1.34 ab	67.77 ± 1.05 b
Long2 fruit (mm)	92.47 ± 1.50 a	86.07 ± 1.83 ab	82.24 ± 1.16 b
Long3 Chalice (mm)	18.56 ±1.33 a	16.02 ± 0.80 a	14.47 ± 0.98 a
Carpels (n°)	5.73 ± 0.18 a	5.93 ± 0.18 a	6.20 ± 0.14 a
Peel weight (g)	123.80 ± 5.90 a	116.34 ± 5.76 a	115.73 ± 8.84 a
Peel thickness (mm)	4.81 ± 0.17 a	4.34 ± 0.23 a	4.61 ± 0.36 a
Seed yield (%)	54.22 ± 0.80 a	56.21 ± 1.44 a	52.17 ± 1.43 a

Different letters within the rows indicate significant differences by Tukey’s test (*p* ≤ 0.05).

**Table 2:** General ANOVA results for the metabolomic leaves analysis, where the compounds with * presented significant differences and “ns” (not significative) were not.

	Main Effects		
Compound	A: Substrate	B: Phenological	INTERACTION: A × B
*Amino acids*			
GABA	*	ns	ns
Alanine	ns	*	ns
Valine	ns	*	ns
Arginine	*	*	ns
Leucine	*	*	ns
Tryptophan	*	*	*
Threonine	ns	*	ns
Aspartate	ns	ns	ns
Phenylalanine	ns	*	ns
Glutamine	ns	*	*
Glutamate	ns	*	ns
*Organic Acids and sugars*			
Formate	ns	*	ns
Acetate	ns	*	*
Malate	ns	*	*
Fumarate	ns	*	ns
Succinate	*	*	ns
Citrate	*	*	*
Sucrose	ns	*	*
Fructose	*	*	*
Glucose	*	*	*
UDP-Sugar	ns	*	ns
Myo-inositol	ns	*	ns
*Secondary Metabolites and others*			
Choline	*	*	ns
Trigonelline	ns	*	ns
Adenine	ns	*	ns
Ferulate	*	*	ns
Gallate	ns	*	ns
Epicatechin	*	*	ns
Vanillate	*	*	ns

**Table 3:** Leave metabolomic analysis results by substrates. The results presented are the mean values ± SE.

	Culture Media		
Compound (mM)	PS0	PS50	PS100
*Amino acids*			
GABA	0.06 ± 0.006 a	0.10 ± 0.007 b	0.08 ± 0.009 ab
Alanine	0.06± 0.003 a	0.07 ± 0.004 a	0.06 ± 0.002 a
Valine	0.03 ± 0.002 a	0.03 ± 0.003 a	0.03 ± 0.001 a
Arginine	0.30 ± 0.03 a	0.47 ± 0.03 b	0.36 ± 0.02 a
Leucine	0.04 ± 0.004 a	0.05 ± 0.005 a	0.05 ± 0.005 a
Tryptophan	0.09 ± 0.01 a	0.14 ± 0.02 b	0.14 ± 0.02 b
Threonine	0.05 ± 0.004 a	0.06 ± 0.0030 a	0.05 ± 0.002 a
Aspartate	0.11 ± 0.009 a	0.12 ± 0.006 a	0.10 ± 0.009 a
Phenylalanine	0.07 ± 0.003 a	0.06 ± 0.002 a	0.07 ± 0.004 a
Glutamine	0.24 ± 0.03 a	0.24 ± 0.02 a	0.20 ± 0.01 a
*Organic acids and sugars*			
Glutamate	0.32 ± 0.02 a	0.34 ± 0.01 a	0.29 ± 0.02 a
Formate	0.02 ± 0.002 a	0.02 ± 0.001 a	0.02 ± 0.002 a
Acetate	0.06 ± 0.007 a	0.06 ± 0.006 a	0.07 ± 0.01 a
Malate	0.33 ± 0.03 a	0.36 ± 0.02 a	0.36 ± 0.04 a
Fumarate	0.14 ± 0.01 a	0.12 ± 0.02 a	0.11 ± 0.01 a
Succinate	0.02 ± 0.002 a	0.02 ± 0.001 a	0.02 ± 0.001 a
Citrate	0.07 ± 0.007 a	0.10 ± 0.01 a	0.09 ± 0.01 a
Sucrose	1.18 ± 0.06 a	1.12 ± 0.04 a	1.09 ± 0.04 a
Fructose	1.29 ± 0.08 a	1.57 ± 0.13 a	1.28 ± 0.09 a
Glucose	2.15 ± 0.26 a	3.34 ± 0.35 b	2.72 ± 0.24 ab
UDP-sugar	0.12 ± 0.01 a	0.12 ± 0.01 a	0.10 ± 0.01 a
Myo-inositol	0.73 ± 0.12 a	0.85 ± 0.12 a	0.68 ± 0.07 a
*Secondary Metabolites and others*			
Choline	0.06 ± 0.006 a	0.08 ± 0.005 a	0.08 ± 0.005 a
Trigonelline	0.03 ± 0.001 a	0.03 ± 0.003 a	0.03 ± 0.003 a
Adenine	0.01 ± 0.001 a	0.01 ± 0.001 a	0.01 ± 0.001 a
Ferulate	0.30 ± 0.02 a	0.53 ± 0.01 b	0.61 ± 0.02 c
Gallate	0.75 ± 0.07 a	0.77 ± 0.06 a	0.72 ± 0.06 a
Epicatechin	0.21 ± 0.01 b	0.10 ± 0.01 a	0.10 ± 0.01 a
Vanillate	0.18 ± 0.01 b	0.08 ± 0.005 a	0.08 ± 0.005 a

Different letters within the rows indicate significant differences by Tukey’s test (*p* ≤ 0.05).

**Table 4:** Metabolomic analysis for three phenological stages (flowering period (FP), fruit development (DF), and postharvest (PostR)) The results show are the mean values in mM ± SE.

	Phenological Stage		
Compound (mM)	Flowering Period (FP)	Fruit Development (DF)	Postharvest (PostR)
*Amino acids*			
GABA	0.08 ± 0.008 a	0.07 ± 0.008 a	0.09 ± 0.009 a
Alanine	0.06 ± 0.002 a	0.06 ± 0.002 a	0.07 ± 0.002 b
Valine	0.02 ± 0.001 a	0.03 ± 0.002 a	0.04 ± 0.003 b
Arginine	0.32 ± 0.03 a	0.32 ± 0.02 a	0.49 ± 0.04 b
Leucine	0.02 ± 0.001 a	0.06 ± 0.003 b	0.06 ± 0.003 b
Tryptophan	0.18 ± 0.01 c	0.07 ± 0.006 a	0.12 ± 0.01 b
Threonine	0.05 ± 0.002 a	0.05 ± 0.003 a	0.06 ± 0.004 b
Aspartate	0.13 ± 0.008 b	0.10 ± 0.009 a	0.12 ± 0.007 ab
Phenylalanine	0.06 ± 0.004 a	0.07 ± 0.004 ab	0.07 ± 0.005 b
Glutamine	0.18 ± 0.02 a	0.19 ± 0.02 a	0.31 ± 0.02 b
*Organic acids and sugars*			
Glutamate	0.37 ± 0.02 b	0.32 ± 0.02 ab	0.26 ± 0.01 a
Formate	0.01 ± 0.001 a	0.03 ± 0.001 b	0.01 ± 0.001 a
Acetate	0.11 ± 0.008 b	0.04 ± 0.002 a	0.04 ± 0.006 a
Malate	0.52 ± 0.03 c	0.21 ± 0.02 a	0.32 ± 0.02 b
Fumarate	0.08 ± 0.005 a	0.15 ± 0.01 b	0.16 ± 0.01 b
Succinate	0.02 ± 0.001 b	0.01 ± 0.001 a	0.01 ± 0.001 a
Citrate	0.12 ± 0.008 c	0.05 ± 0.007 a	0.09 ± 0.009 b
Sucrose	1.22 ± 0.02 b	0.96 ± 0.02 a	1.22 ± 0.06 b
Fructose	1.60 ± 0.07 b	0.94 ± 0.04 a	1.65 ± 0.09 b
Glucose	3.50 ± 0.23 b	1.55 ± 0.14 a	3.21 ± 0.32 b
UDP-sugar	0.12 ± 0.005 b	0.08 ± 0.008 a	0.14 ± 0.02 b
Myo-inositol	0.58 ± 0.04 a	0.47 ± 0.02 a	1.27 ± 0.12 b
*Secondary Metabolites and others*			
Choline	0.10 ± 0.002 b	0.06 ± 0.003 a	0.05 ± 0.003 a
Trigonelline	0.03 ± 0.002 b	0.02 ± 0.001 a	0.02 ± 0.001 a
Adenine	0.02 ± 0.001 b	0.01 ± 0.001 a	0.01 ± 0.001 a
Ferulate	0.50 ± 0.04 a	0.50 ± 0.03 a	0.43 ± 0.02 a
Gallate	0.45 ± 0.02 a	0.86 ± 0.04 b	0.95 ± 0.04 b
Epicatechin	0.12 ± 0.02 a	0.16 ± 0.01 a	0.13 ± 0.02 a
Vanillate	0.10 ± 0.01 a	0.13 ± 0.02 a	0.12 ± 0.01 a

Different letters within the rows indicate significant differences by Tukey’s test. (*p* ≤ 0.05).

**Table 5:** Analysis of variance components (%) for the leaves metabolomic analysis. For each of the variables analyzed, the amount of variability of each of the factors (substrate and phenological phase) is shown.

Compound	Phenological Stage	Substratum	Mistake
*Amino acids (%)*			
Arginine	20.62	0.00	79.38
Leucine	0.00	38.50	61.50
Tryptophan	5.22	34.82	59.97
Threonine	33.65	24.43	41.92
Aspartate	77.48	7.31	15.21
Phenylalanine	33.45	29.29	37.27
Glutamine	20.23	2.04	77.73
Arginine	10.17	0.00	89.83
Leucine	13.20	8.61	78.20
Tryptophan	36.87	17.40	45.73
*Organic acids and sugars (%)*			
Glutamate	27.53	0.73	71.74
Formate	67.47	1.75	30.78
Acetate	75.72	8.79	15.49
Malate	72.58	4.32	23.11
Fumarate	46.09	3.00	50.90
Succinate	33.97	5.55	60.48
Citrate	46.88	18.93	34.19
Sucrose	40.35	18.56	41.09
Fructose	55.52	30.04	14.44
Glucose	45.84	26.86	27.30
UDP-sugar	27.85	0.00	72.15
Myo-inositol	66.49	2.06	31.45
*Secondary metabolites and others (%)*			
Choline	74.42	12.19	13.39
Trigonelline	41.70	0.30	57.99
Adenine	53.56	0.55	45.89
Ferulate	0.00	86.45	13.55
Gallate	78.59	0.00	21.41
Epicatechin	0.00	70.72	29.28
Vanillate	0.00	71.38	28.62

## Data Availability

Data is contained within the article.
